# Cancer of the Inferior Lip and Corresponding Maxillary Bone; Cure after Two Operations, Performed at Different Periods

**Published:** 1844-05

**Authors:** Martial Dupierris

**Affiliations:** Havana


Art. V.—*Cancer of the inferior lip and corresponding maxillary bone: Cure after two operations, performed at different periods.* By Martial Dupierris, M.D., of Havana.

D. Francisco Alvarez, born at Cadiz, aged 50, an ex-officer of the custom-house in Havana, of bilious-sanguine temperament, and endowed with a pretty strong constitution, consulted me, on the 19th July, 1843, for an affection of the lower lip, which was attended with horrible suffering.

I learned that a small pimple had first made its appearance a short distance from the left commissure of the lips, and had been followed very soon by an ulcer attended with lancinating pains, which gave the patient not a moment's repose. This ulcer extended towards the right side, involving a large portion of the lip; it also attacked the cheek internally. Divers means which were prescribed by a professor of this city, failed to allay the sufferings of the patient, or check the progress of the disease; on the contrary, the pains augmented in severity, the ulcer enlarged daily, and bled upon the slightest touch.

When the patient presented himself to me, the surface ot the ulcer was grayish and unequal, the edges everted and hard, and the discharge from it of an ichorous nature. It had destroyed a large portion of the soft parts comprised between two lines: one drawn on the right side, from the free border of the lip, about ten lines from the commissure, to the centre of the hyoid bone; the other, beginning at the left commissure, and pursuing a similar direction until it joined the first; the triangular space thus marked out, presented the exact form of the ulcer externally. Internally it had invaded the left cheek as far as the anterior edge of the masseter muscle, involving all of the tissues except the integuments. The first two molars of the right side were wanting in the lower jaw. The tissues adjacent to the ulcer were hard, red, and painful.

The patient possessed a good deal of courage, and having been attended by me 15 years before, in the Island of Porto Rico, during an attack of typhoid fever, which came very near proving fatal to him, he begged me earnestly to do something to rid him of his fearful malady. Encouraged by such a disposition on the part of the patient, I did not hesitate to undertake the following operation, which was performed on the 23d of July, 1843, with the assistance of Dr. Coronado, licientates Herrera, Cabellero, Otero, and one of my students, and in the presence of a few friends of the patient.

The patient being seated on a firm chair, and the facial arteries, as they pass over the jaw, compressed by an assistant, I made an incision, beginning at the free edge of the lip, three lines from the right commissure, and proceeding downwards to the middle of the os hyoides; a similar incision, commencing at the commissure of the left side, joined the first at an acute angle; a third was carried from the left commissure, in the direction of the superior dental arch, to the anterior edge of the masseter, and from the extremity of the latter I made a fourth, which circumscribed all the affected parts, and became continuous with the second; lastly, a fifth incision was carried from the right commissure to the masseter of the same side. Torsion was applied to the arteries, which bled profusely. After having dissected out carefully all the morbid parts, I discovered that the bone was slightly affected with cancer; the periosteum had disappeared over a space of three lines towards the symphysis, on the left side. After having scraped the bone until it presented a healthy aspect, I cleansed the flaps, approximated that of the left side as closely as possible to the one on the right, raising up the former towards the flap made by the transverse incision of the cheek, to which I united it by means of eight points of the twisted suture. The mucous membrane was retained in contact with the integuments of the free edge of the artificial lip by five points of the interrupted suture.

After the operation, Alvarez had fever which required bloodletting and a continuance of the diet that had been prescribed. Cicatrization occurred every where except at the upper part of the junction of the two lateral flaps; this point remained open in spite of every thing I could do. Twenty days after the operation, the artificial lip might have been said to be natural, as nothing could be perceived save three linear cicatrices, and the ulceration just mentioned, which, three months afterwards, from its character and extent required an operation of far greater severity, which I shall now describe.

At the bottom of the wound, something hard could be felt with a probe; this was the maxillary bone affected with cancer, or rather the point where this terrible disease renewed its ravages. I therefore prepared the constitution of my patient in a suitable manner, and fixed the operation for the 17th October. Dr. Coronado, licentiates Herrera, Muezes, &c., and one of my pupils, kindly lent me their aid a second time.

The patient being fixed firmly to a seat, incisions were made through the soft parts in the same direction as at the first operation, only they were prolonged as far as the thyroid cartilage, in order to remove all that might be found affected by the rapid progress of ulceration within a few days. The flaps being dissected backwards, exposed about six inches of the lower jaw; the third left molar was extracted, and the bone divided by means of the chain-saw; the second molar of the right side being absent, the saw was applied at that point, but the extremity not presenting a healthy aspect, about two lines more were removed, which left the bone perfectly healthy in appearance. A loop of thread was then passed through the superior part of the genio-glossi muscles, and by means of a strait bistoury, I removed that portion of bone isolated by the saw, carrying the knife close to the concave surface.

In the course of the operation, torsion was applied to four arteries, and hemorrhage was checked without difficulty by lotions of vinegar and water. The wound was large and hideous; six needles and the twisted suture were applied at proper distances, and the mucous membrane was fixed to the skin by the interrupted suture, as in the first operation. The integuments were by no means abundant, but were sufficient to close the great opening necessarily made in order to de stroy the malady.

The patient went to bed; an anti-spasmodic mixture was given him, with directions as to diet. Acute pains, and very considerable excitement supervened; these were allayed somewhat by v. s. to 16 ounces. Compresses wet with resolvent lotions were kept constantly applied. On the third day, there was difficult deglutition, and obstructed respiration; a tumor, the size of a pigeon's egg, had formed at the lower part of the cicatrized wound, and when opened, gave exit to a considerable quantity of mucus slightly tinged with blood; a piece of lint was inserted in the opening to prevent a re-accumulation of matter.

On the 23d, the two lower needles were removed, allowing the threads to remain; on the 26th, the other needles were likewise withdrawn, the cicatrization being complete save at the inferior part where the tent was introduced. This was removed some days afterwards, and nothing else occurred. In a few days, we could perceive that the extremities of the bone were losing their hardness, and becoming covered with vascular granulations, which on each side adhered to the labial mucous membrane. A month subsequently, a pretty firm substance had formed in the interval between the two ends of the bone; but unfortunately this work of nature was suddenly arrested, and the tissue is not sufficiently firm to consolidate the parts like the lost bone. I can attribute this arrest to nothing but the advanced age of the patient.

Remarks.—I have not dwelt minutely on the attention required by these two operations, because nothing extraordinary occurred. I will only observe that the rapidity with which the wounds cicatrized, and the absence of any accident subsequent to these important operations, are due, it seems to me, to the method which I employed, a method which Professor Serre, of Montpelier, has so well explained. It is easy to see that any other mode of autoplasty, would not have enabled me to perform the second operation with a chance for such rapid and brilliant success. This is particularly evident from the present state of Alvarez who, at this time (Feb. 15), four months after the second operation, not only enjoys good health, but moreover does not present the least appearance of a return of the affection. It is true, the deformity is striking; but was it possible to avoid it after two operations of so grave a character? No one will presume to say so. Hence the patient does not cease to evince his gratitude; and the beautiful results, which the progress of surgery has enabled me to bring about, render me happy in thinking that, for the third time, I have saved the life of an unfortunate man, who has a family dependent on him for support.

The portion of bone removed, and which I preserve, is porous (*criblée*) for an extent of three inches, nothing being left but a thin lamella of the posterior plate.

I seize the opportunity to mention here that for twenty years I have used cold water, acidulated with vinegar, to deterge wounds, whether produced by violence, or in the course of numerous operations performed by me, and have never had to deplore the occurrence of tetanic symptoms, nor the absorption of air by the veins, although the former of these affections is very common in the countries where I have exercised my calling. I shall forbear adding any explanation of the mode of action of this remedy, which I think may be considered as a true prophylactic against the development of tetanus; yet, in my opinion, a satisfactory explanation could be given.

Havana, February 15, 1844.

